# Impact of predictive selection of LbCas12a CRISPR RNAs upon on‐ and off‐target editing rates in soybean

**DOI:** 10.1002/pld3.627

**Published:** 2024-08-16

**Authors:** Linda Rymarquis, Chenxi Wu, Diane Hohorst, Miguel Vega‐Sanchez, Thomas E. Mullen, Vijetha Vemulapalli, Douglas R. Smith

**Affiliations:** ^1^ Bayer Crop Science Chesterfield Missouri USA; ^2^ SeQure Dx Waltham Massachusetts USA

**Keywords:** genome editing, gRNA design, LbCas12a

## Abstract

Clustered regularly interspaced short palindromic repeats (CRISPR) technology has revolutionized creating targeted genetic variation in crops. Although CRISPR enzymes have been reported to have high sequence‐specificity, careful design of the editing reagents can also reduce unintended edits at highly homologous sites. This work details the first large‐scale study of the heritability of on‐target edits and the rate of edits at off‐target sites in soybean ( *Glycine max* ), assaying ~700 T1 plants each resulting from transformation with LbCas12a constructs containing CRISPR RNAs (crRNAs) predicted to be either “unique” with no off‐target sites or “promiscuous” with >10 potential off‐targets in the soybean genome. Around 80% of the on‐target edits observed in T0 plants were inherited in the T1 generation, and ~49% of the total observed on‐target edits in T1 were not observed at T0, indicating continued activity of LbCas12a throughout the life cycle of the plant. In planta editing at off‐target sites was observed for the Promiscuous but not the Unique crRNA. Examination of the edited off‐target sites revealed that LbCas12a was highly tolerant to mismatches between the crRNA and target site in bases 21–23 relative to the start of the protospacer, but even a single mismatch in the first 20 nt drastically reduced the editing rate. In addition, edits at off‐target sites have lower inheritance rates than on‐target edits, suggesting that they occur later in the plant's lifecycle. Plants with a desired on‐target edit and no off‐target edits could be identified in the T1 generation for 100% of the T0 plants edited with the Unique crRNA compared with the 65% of T0 plants edited with the Promiscuous crRNA. This confirms that proper crRNA selection can reduce or eliminate off‐target editing. Even when potential off‐target sites are predicted, plants containing only the intended edits can still be identified and propagated.

## 1 INTRODUCTION

With the rapidly growing global population and the need for increased sustainability in agriculture (Brain et al., 2023), there is an escalating need for the use of new breeding technologies to bring higher‐yielding, climate‐ and pest‐resilient crops to market in a more efficient and expedited manner. Genome editing stands out as a technology for its potential to rapidly and precisely modify crop genomes to improve agronomic and other traits. Clustered regularly interspaced short palindromic repeats/CRISPR‐associated protein (CRISPR/Cas) is one of the genome editing technologies available, enabling precise genetic modifications in diverse organisms through a three‐step process (Asmamaw & Zawdie, 2021; Jinek et al., 2012): (1) The guide RNA (gRNA) or CRISPR RNA (crRNA), acts as a molecular “homing device” guiding the CRISPR/Cas nuclease as it navigates the crop genome for protospacer adjacent motifs (PAMs) and a complementary DNA target of interest; (2) Binding of the CRISPR/Cas nuclease to the target sequence and introduction of DNA double‐strand breaks (DSBs); (3) Exploiting the endogenous DNA repair mechanisms of plants, such as homology‐directed repair or non‐homologous end joining, to generate genetic variation or edits.

Because recognition of the target sequence is homology‐based, off‐target edits, which occur when a locus with high, but not perfect, sequence homology to the gRNA/crRNA sequences is edited, can be generated through CRISPR/Cas (Graham et al., 2020). Generation of off‐target edits with the CRISPR/Cas system is generally considered rare in plants, as assessed by a plethora of studies using multiple CRISPR enzymes. Modrzejewski et al. (2019) took a meta‐analysis approach and analyzed 220 studies that have explored off‐target edits from Cas9 and Cas12a in over 30 different plant species and subspecies. Off‐target edits were detected in only 3% of the 1738 different potential off‐target sites investigated in the 211 studies analyzed. These results have been confirmed by more recent studies for Cas9 (Sagarbarria et al., 2023; Wang et al., 2021), as well as additional independent studies that reported high genome editing specificity for the Cas12a nuclease (Bernabé‐Orts et al., 2019; Li et al., 2019; Tang et al., 2018; Zhang et al., 2023). Although off‐target edits are very rare in plants, it is recommended to assess and minimize the off‐target potential of gRNA/crRNAs, especially in work involving the commercial development of gene‐edited crops, to ensure the integrity of edited products.

Genome‐wide off‐target profiling can be done both in vitro and in vivo (thoroughly reviewed by Manghwar et al., 2020; Naeem et al., 2020; Guo et al., 2023; Jiménez & Crosetto, 2023). For in vitro off‐target tools, synthetic or genomic DNA is incubated with Cas/gRNA ribonucleoprotein (RNP) complexes in a test tube, and cleavage sites are identified through next‐generation sequencing (e.g., CIRCLE‐seq, OligoNucleotide Enrichment and sequencing (ONE‐seq) (Petri et al., 2021; Tsai et al., 2017). However, in vitro tools are prone to false‐positive results and do not capture CRISPR activity in cells or in intracellular environments (Atkins et al., 2021; Kim & Kim, 2018; Sagarbarria et al., 2023). Therefore, in vivo methods using DNA DSBs as a proxy for genome‐wide off‐target edits in living cells or organisms are needed to validate the in vitro results. These methods either directly label or fix DSBs (e.g., genome‐wide unbiased identification of DSBs enabled by sequencing [GUIDE‐seq], Malinin et al., 2021), measure the repair outcomes of the DSBs, such as chromosomal translocations by high‐throughput genome‐wide translocation sequencing (HTGTS, Frock et al., 2015), or detect point mutations, small insertions, and deletions through methods such as verification of in vivo off‐targets (VIVO, Akcakaya et al., 2018).

The scientific standard based on numerous studies evaluating off‐target edit rates of CRISPR nucleases in plants is that proper design of gRNAs with bioinformatic tools to limit off‐target editing potential leads to no detectable levels of off‐target edits in planta (Aksoy et al., 2022; Gerashchenkov et al., 2020; Graham et al., 2020; Manghwar et al., 2020). Previous studies using Cas9 have revealed major characteristics of an effective crRNA design (Doench et al., 2014, 2016; Kuan et al., 2017; Modrzejewski et al., 2020), including (1) the number of mismatches in both the protospacer sequence and PAM sites; (2) the position of mismatches (e.g., the PAM‐proximal seed region); (3) the nucleotide composition; and (4) the crRNA secondary structure. The importance of such features varies by the different enzyme types (i.e., Cas9 vs. Cas12a), especially when comparing in vitro data to in vivo data (Jones et al., 2021).

In the present study, a strategy similar to that used to validate gRNA design for Cas9 in both plant and animal systems (Akcakaya et al., 2018; Young et al., 2019) was used with LbCas12a to determine how the above‐mentioned crRNA selection and design features impact editing efficiency and inheritance at on‐ and off‐target sites. Our crRNA validation contained two steps: (1) an initial in vitro “discovery” step using NoteSeQ, which is a variant of ONE‐seq (Petri et al., 2021), that measures cleavage by the LbCas12a RNP on a synthesized DNA for a panel of computationally identified potential off‐target sites; and (2) an in vivo “confirmation” step that examined the potential off‐target sites identified by NoteSeQ in soybean T1 plants using amplicon sequencing. We compared editing rates between the Unique crRNA (designed with no predicted off‐target edit potential) and the Promiscuous crRNA (predicted to have >10 off‐target edits) both in vitro and in planta for LbCas12a. Inheritance of edits at the on‐target loci was tracked from the T0 to the T1 generation, and the percent of T1 progeny was monitored for the presence, if any, of off‐target edits. We also discuss how the learnings from this study can be used to optimize crRNA design tools that avoid off‐target activities.

## 2 METHODS AND MATERIALS

### 2.1 crRNA identification

**FIGURE 1:**
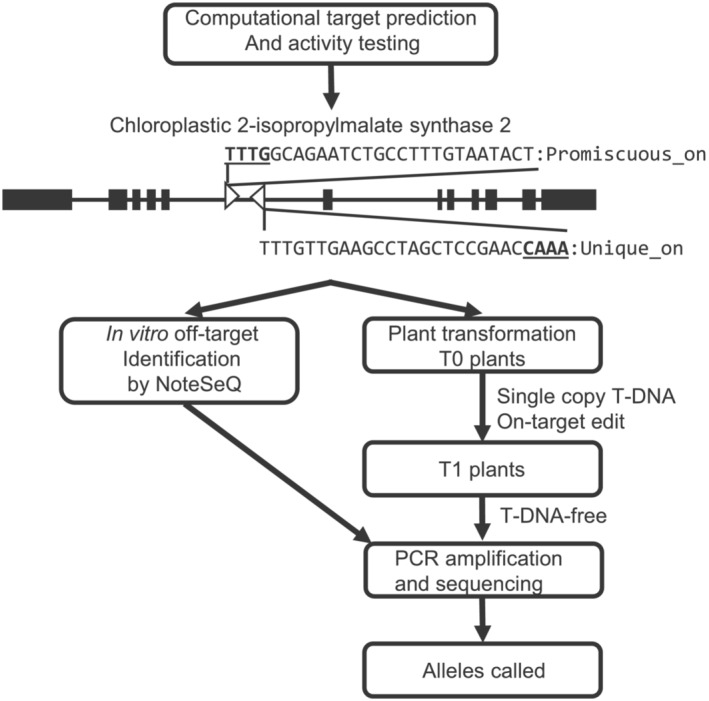
Experimental overview for on‐ and off‐target analysis. In vitro and in planta experiments were done in parallel. The chloroplastic 2‐isopropylmalate synthase 2 gene is shown in the 5′ to 3′ orientation. The black boxes represent the exons, while the white triangles indicate the location and orientation of the Promiscuous_on and Unique_on target sites. The protospacer adjacent motif (PAM) sequences are underlined and bolded. Plants were transformed with a plasmid expressing the LbCas12a nuclease and either the Unique, Promiscuous or no crRNA. The NoteSeQ experiments informed the assay panel for off‐target analysis.

LbCas12a target sites were predicted genome‐wide using Python scripts. In short, all TTTV PAM sites were identified in the A3555 soybean genome, and the 23 nucleotides (nt) downstream were pulled. For computational off‐target analysis, target sites were compared with the genome with up to three mismatches using the Bowtie short read aligner (Langmead et al., 2009) to identify additional on‐targets and potential off‐target sites. For off‐target analysis, TTTN, NTTN, TTCA, and TTCC were considered valid PAMs. crRNAs that only perfectly matched the genome once were clustered into 1 kb bins. Bins predicted to have >5 target sites that were targeted by a combination of crRNAs with no predicted off‐targets and >10 off‐target sites were overlapped with gene models to identify those in non‐essential/duplicated genes to avoid causing any phenotypic differences in edited plants that could cause selection bias. The chloroplastic 2‐isopropylmalate synthase 2 gene was chosen because it is part of a gene family. It has at least 4 copies, with another 8 genes being listed as probably 2‐isopropylmalate synthase in the NCBI database. In addition, de Kraker et al. (2007) showed that knocking out one copy of the gene in Arabidopsis did not impact plant health. Six crRNAs, 4 with predicted off‐targets and 2 without, targeting chloroplastic 2‐isopropylmalate synthase 2, were tested for activity via plant transformation to pick a single crRNA, each meeting the “promiscuous” or “unique” criteria that had similar editing activity to each other for off‐target testing in vitro for large scale in planta testing (see methods below, Table S1). Hereafter, these chosen crRNA will be called Promiscuous or Unique crRNA. The on‐target sites are shown in Figure 1, and the protospacers are the bolded sequences in Table S1.

### 2.2 In vitro off‐target analysis

The sequence of protospacers from the Unique and Promiscuous crRNAs was sent to SeQure Dx (Waltham, MA, USA) for NoteSeQ analysis. Comprehensive genome‐wide sets of candidate off‐target sites with up to 8 mismatches and no bulges, or up to 5 mismatches and 1 DNA or RNA bulge of size 1 or 2, were generated for each guide using Cas‐Designer (Park et al., 2015) with the A3555 soybean genome sequence. The sequences were incorporated into a set of custom oligonucleotide sequences, wherein each candidate off‐target site was flanked by a unique pair of barcodes and constant regions. The oligonucleotide design and subsequent enzymatic steps described below followed the procedures described by Petri et al. (2021). Oligonucleotide pools representing each sequence were synthesized (Agilent Technologies, Santa Clara, CA, USA) and amplified by limited cycle PCR using Phusion polymerase (New England Biolabs, Ipswich, MA, USA). The resulting library of double‐stranded molecules was subjected to in vitro editing using the LbCas12a protein (New England Biolabs, Ipswich, MA, USA) and the appropriate crRNA (IDT, Coralville, IA, USA). Cleavage reactions were performed in triplicate at two RNP/DNA ratios, 10/1 and 1/1. The resulting cleaved molecules were ligated to adapters, amplified, indexed, and sequenced on an Illumina instrument. The sequencing data for each run was analyzed using a custom analysis pipeline with a cleavage site window of 6 on the PAM side and 8 on the protospacer side. The library barcodes were used to identify the cleaved halves corresponding to each candidate off‐target site. A score was calculated for each site by taking the read counts for a given site and dividing them by the read counts of the on‐target site. Background was set to 10 reads or the point at which the average read counts leveled out when graphing the counts for all targets, whichever was higher. For Promiscuous crRNA, the background was 10 reads, whereas for Unique crRNA, the background was 25 reads. To be considered an off‐target, either the 10:1 or 1:1 read counts had to be above background for at least 1 genomic locus.

### 2.3 Plasmid construction and plant transformation

All plasmids contained an LbCas12a expression cassette and an aadA marker cassette. The LbCas12a expression cassette consisted of a codon‐optimized LbCas12a coding region driven by the Medicago ubiquitin2 constitutive promoter and followed by a Medicago AC145767 terminator. The aadA cassette, driven by an Arabidopsis actin7 promoter and nos terminator, was used as the selection marker. The crRNA cassettes for the Unique and Promiscuous plasmids contained a soybean U6 pol III promoter, GTCC as the transcription start site, LbCas12a repeat (TAATTTCTACTAAGTGTAGAT), a 23‐nt protospacer (Figure 1), followed by the LbCas12a repeat, and a 9‐nt poly(T). The original crRNA testing plasmid used to generate Table S1 contained two multiplex crRNA cassettes. The first consisted of the soybean U6 pol III promoter, GTCC as the transcription start site, LbCas12a repeat, protospacer 1 (Table S1), repeat, protospacer 2, repeat, protospacer 3, repeat, and 9 nt poly(T). The second crRNA cassette had the same structure as protospacers 4–6, except it used a soybean 7SL promoter. Vectors were transformed into A3555 as previously described (Ye et al., 2023).

### 2.4 Transgene copy number assay

DNA was extracted from the first unifoliate leaf from the transformed events (T0) as described in Kouranov et al. (2022), except that the cleared lysate volume and isopropanol volumes were increased to 125 uL and 80 uL were used for DNA elution. Primers used can be found in Table S2. The copy number of the AC145767 terminator and aadA marker were assayed by standard TaqMan PCR using the Mt.Ac145767 3'UTR and aadA primer sets, respectively. TaqMan GTxpress Master Mix (ThermoFisher Scientific, Waltham, MA, USA) was used according to the manufacturer's protocol. Thermal cycling was performed on an Applied Biosystems (Waltham, MA, USA) ViiA 7 Real‐Time PCR System.

### 2.5 T0 assay for editing

The genomic region containing both the Promiscuous and Unique on‐target loci (Promiscuous_on and Unique_on) was amplified using the Glyma.20G245300 primer set (Table S2), sheared, and libraries prepared for Illumina sequencing (Illumina San Diego, CA, USA) as described in Kouranov et al. (2022). Reads were trimmed using trimmomatic (Bolger et al., 2014) and mapped to their respective locus using glsearch (Pearson & Lipman, 1988). Edits were called if sequence overlapped the 23 nt region bound by the protospacer of LbCas12a crRNA. Sequence variants with >10% of the sequencing reads covering the target site were considered edited alleles. Editing rates were calculated by taking the number of edited plants/total plants assayed*100.

### 2.6 T1 assays for LbCas12a

To detect the absence of the LbCas12a nuclease in T1 progeny, an automated high‐throughput seed chipper was used to nondestructively sample the T1 seeds. A small amount of cotyledon tissue was collected from each seed into 96‐well plates. DNA was isolated and genotyped using qualitative endpoint TaqMan assays for the right border (GM_oRB_ext116 primer set, Table S2). TaqPath ProAmp master mix (ThermoFisher Scientific, Waltham, MA USA) was used according to the manufacturer's protocol. Thermal cycling was performed on an Applied Biosystems GeneAmp PCR system 9700 (Waltham, MA, USA) and fluorescence measurement was performed by a Tecan Spark microplate reader (Tecan, Männedorf, Switzerland).

### 2.7 T1 editing assays and analysis

DNA was extracted from 2‐hole punches from the first unifoliate leaf using Magmax (Macharey Nagel, Allentown, PA, USA) according to the manufacturer's instructions. DNA from 689 Unique plants, 694 Promiscuous plants and 389 Control plants was sent to AgriPlex Genomics (Cleveland, OH, USA) for PlexSeq assay creation and sequencing. The AgriPlex PlexSeq workflow multiplexes all assays and delivers trimmed data. Fastq files AgriPlex generated are available in NCBI under Bioproject PRJNA1095968. An overview of the analysis pipeline is in Figure S1. To isolate reads from individual genomic loci, kmers (Table S3) specific to each locus were matched to the reads. Reads that matched both kmers for a corresponding locus were extracted and mapped to their respective locus using glsearch (Pearson & Lipman, 1988). Edits were called if sequence variants overlapped the 23 nt region that binds the protospacer of the LbCas12a crRNA. Due to the high homology between the various genomic loci, template switching was observed in plants transformed with all three plasmids. This caused substitutions in the target site, which mapped it to another locus. Reads derived from template switching were removed. Due to some primer sets detecting two loci in one assay, edit allele detection was lowered to having ≥5 reads associated with the edited allele and having ≥5% of the total reads associated with the target site. Loci chr19_12052265 and chr9_24400619 were indistinguishable except for the single nucleotide polymorphism (SNP) in the guide region, and allele calls were manually separated based on this SNP. Also, targets chr17_13932647 and chr14_41419116 were indistinguishable except for one SNP 10 nt from the guide, and because there were no edits observed, they were reported as a single locus under chr14_41419116. Chi‐squared tests were performed with a *p*‐value of .01 to determine deviation from standard Mendelian inheritance.

## 3 RESULTS

Figure 1 details the experimental overview, which can be broken into three parts: (1) selection of the Unique and Promiscuous crRNAs; (2) in vitro testing by NoteSeQ to discover potential off‐target loci; and (3) in planta validation to determine editing rates at on‐ and off‐target loci and heritability patterns. To find suitable crRNAs with either no predicted off‐targets (“unique”) or with >10 predicted off‐targets (“promiscuous”), a whole genome search was conducted to find clusters of LbCas12a target sites that overlapped a non‐essential gene and had a combination of crRNAs meeting the “unique” or “promiscuous” criteria. Six crRNAs targeting chloroplastic 2‐isopropylmalate synthase 2, corresponding to the public gene id Glyma.20G245300.3 on the William 82.v1 genome, were tested for editing activity (Table S1) in T0 plants. A single crRNA, each meeting the “unique” criteria, hereafter called Unique crRNA or the “promiscuous” criteria, hereafter called Promiscuous crRNA, that had similar levels of editing activity to each other (80% and 74%, respectively, Table S1), was selected for further in vitro and large‐scale in planta work.

**FIGURE 2:**
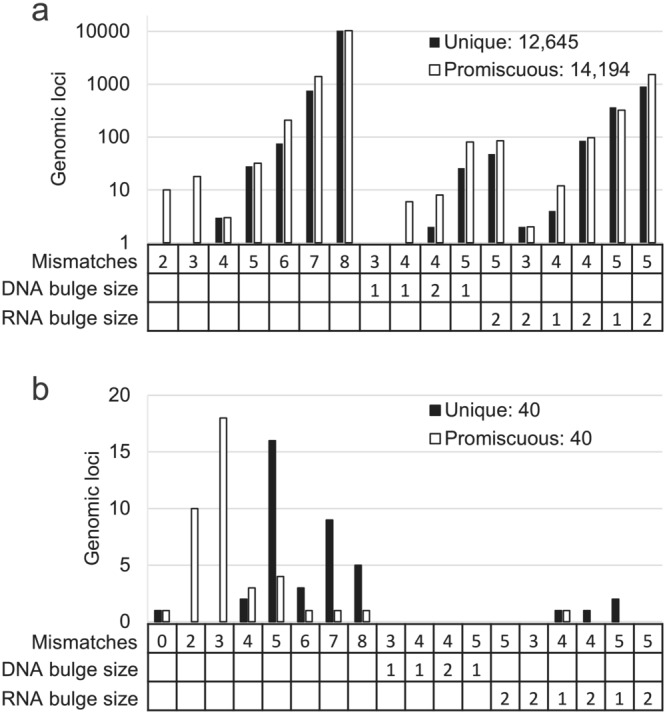
In vitro off‐target analysis by NoteSeQ. (a) The off‐target panel created for NoteSeQ analysis showing the different combinations of mismatches and bulges present for examined genomic loci. (b) Number of on‐ and off‐target genomic loci with in vitro evidence of LbCas12a cleavage in the NoteSeq analysis for each combination of mismatches or bulges.

To capture the full range of potential off‐target edits, the Unique and Promiscuous crRNA sequences were sent to SeQure Dx for in vitro NoteSeQ analysis, which is a variant of ONE‐seq (Petri et al., 2021). All genomic loci containing up to a total of 8 mismatches and/or bulges were identified to create panels of synthetic DNA substrates. Both test panels contained greater than 12,000 potential off‐target genomic loci, containing 5937 and 11,074 target sites with distinct sequences for Unique and Promiscuous crRNAs, respectively (Figure 2a). NoteSeQ analysis was run with 2 LbCas12a RNP/DNA concentrations, 10 RNP:1 DNA, and 1 RNP:1 DNA duplex. Each target was given a NoteSeq score by dividing the reads for the target site/reads for the on‐target. After NoteSeQ analysis, 40 genomic loci each were found to be potential off‐target sites for Unique and Promiscuous crRNAs, with the NoteSeQ scores ranging from >1 down to .000237 (Table S4). The 40 genomic loci consisted of 23 and 20 distinct target site sequences for Unique and Promiscuous crRNA, respectively (Figure 2b, Table S4). Each distinct target sequence was named according to their crRNA, followed by “on” for the on‐target sites or a number for the off‐target sites. Each off‐target locus was named according to its genomic coordinates.

**FIGURE 3:**
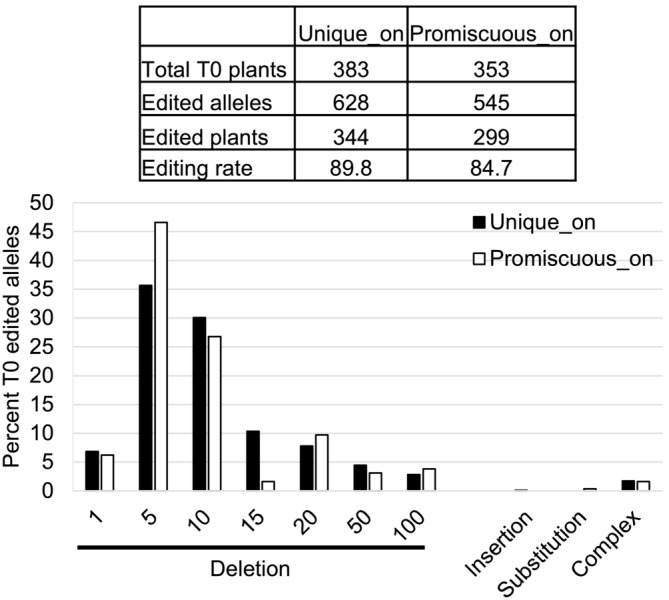
Edit profile at the Unique_on and Promiscuous_on target sites in T0 plants. Size of the deletions were binned and shown as the percent of total edited alleles. The percent edited alleles comprising insertions, substitutions, and complex edits, which contain a combination of deletions, insertions, and substitutions, are also shown.

To test editing at on‐ and off‐target loci in planta, three plasmids were constructed. The Control plasmid contained the LbCas12a cassette and the aadA selectable marker alone, without any crRNA. The Promiscuous and Unique plasmids contained an additional crRNA cassette expressing either Promiscuous or Unique crRNA. These plasmids were transformed into soybean variety A3555, and the resulting plants were tested for transgene copy number. A total of 383 and 353 T0 plants were assayed for edits at the on‐target loci (Unique_on and Promiscuous_on), with 628 and 545 edited alleles identified, respectively (Figure 2a). Deletions were the most prominent editing outcome, with ~80% of the edits being <20 nt in length (Figure 3b).

### 3.1 Inheritance of edits from the T0 generation

**FIGURE 4:**
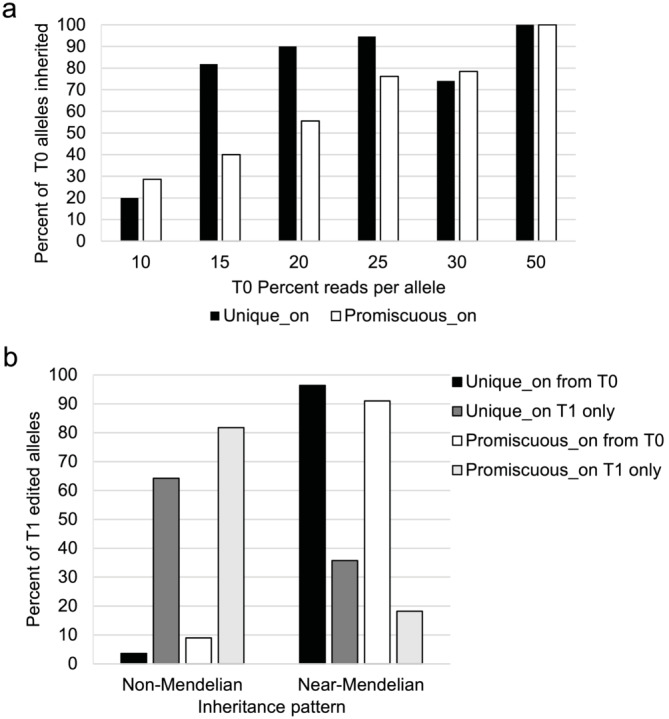
Inheritance of edited alleles at the Unique_on and Promiscuous_on target sites in the T1 generation. (a) The percent T0 alleles observed in the T1 generation as a function of the percent of reads for those alleles in the T0 generation. Results were binned with the lower limit being displayed on the graph. (b) The percent of T1 edited alleles versus the inheritance pattern for edits observed in the T0 generation (from T0) and the edits present only in the T1 generation and not the parental line (T1 only). Non‐Mendelian inheritance pattern is classified as edits found in <20% of the T1 progeny from its T0 parent, whereas near‐Mendelian is in <20% if the T1 progeny from its T0 parent.

To only examine edits generated during the T0 generation and not de novo edits generated in the T1 generation, seed chipping and TaqMan assays were used to identify null segregants that lacked the LbCas12a transgene in T1 seeds. The average null‐segregant rate in T1 seed was 25.5%–34.5%, which is in line with normal Mendelian segregation of the transgene. Unifoliate leaves from between 9 and 12 null‐segregant progeny from 59 Unique, 60 Promiscuous, and 36 Control T0 parental plants were sampled and sent to AgriPlex Genomics for assays. Between 77% and 88% of the on‐target edits observed in the T0 generation were inherited in the T1 generation (Table S5). The percent reads aligning to a particular edited allele in the T0 assay was predictive of its T1 inheritance. Edited alleles having a higher percent of reads in T0 had higher rates of inheritance in the T1 generation (Figure 4a). For example, for T0 edited alleles supported by only 10%–15% of the total reads spanning that target site, only 20%–30% of those alleles were observed in T1 progeny, whereas edits with ≥50% reads in T0 plants inherited near 100% of the time.

To obtain a baseline of the rates of background variation in this experiment, all on‐ and off‐target sites for both Promiscuous and Unique crRNAs were examined in T1 plants transformed with each of the three plasmids. Changes in target sites for which the corresponding crRNA was never expressed in the plant could arise from many processes, such as spontaneous mutation, somaclonal variation, sequencing errors, or potential cross‐contamination between plants/samples (Graham et al., 2020). As expected, no edits for any on‐ or off‐target sites were found in the Control plants. Edits in the Promiscuous on‐ and off‐target sites were only found in the T1 Promiscuous plants. No edited alleles were called for any of the Unique off‐target sites. For the Unique_on locus, edits were found in 86% of the T1 Unique plants and in two (.29%) of the T1 Promiscuous plants. Combining the Control and Promiscuous plants together, out of 1083 plants not expressing the Unique crRNA, .18% had edited alleles called for Unique_on. This suggests that the rate of background variation ranged from 0 to a maximum of .18% for this experiment.

Edits observed in the T1 progeny, but not their T0 parent, comprised 49% of the total edited alleles in the T1 generation for both the Promiscuous and Unique crRNA (Table S5). Because a constitutively expressed Cas12a was used, these edits likely originated from chimeric T0 plants, where the editing occurred after the T0 unifoliate leaf was formed. This is reflected in the percentage of T1 progeny containing the edits (Figure 4b). Edited alleles that were found in ≤20% of progeny deviated from the expected Mendelian inheritance pattern of 75% with a *p*‐value of <.01 and were categorized as having a non‐Mendelian inheritance pattern. Alleles present in <20% of progeny were categorized as near‐Mendelian inheritance pattern. Edits that were inherited from T0 were in an average of 63%–66% of the progeny derived from their T0 parent, with >90% of edits being classified as near‐Mendelian. T1 only edits were in an average of 19%–25% of progeny from their respective T0 parent, with 60%–80% of the T1 only edits falling into the non‐Mendelian category, supporting their chimeric nature.

### 3.2 Editing at the off‐target sites predicted by NoteSeQ

**FIGURE 5:**
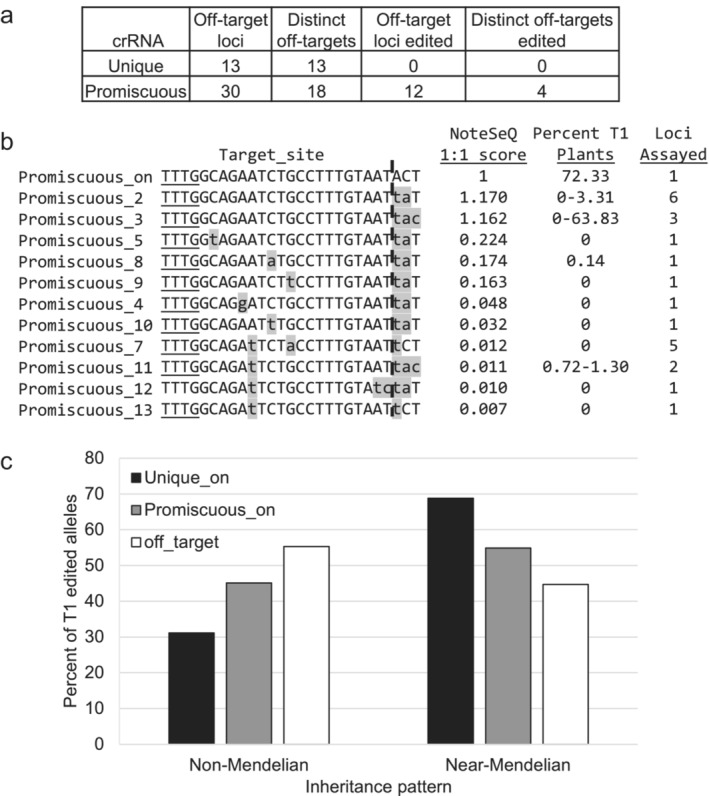
Inheritance of edited on‐ and off‐target sites predicted by NoteSeQ. (a) The number of loci and distinct off‐target sequences predicted by in vitro NoteSeQ analysis that were assayed and edited for each clustered regularly interspaced short palindromic repeats (CRISPR) RNA (crRNA). (b) The location of mismatches and average editing rates for all Promiscuous on‐ and off‐target sites with a 1:1 ribonucleoprotein (RNP)/DNA NoteSeQ score >.001. Mismatches are highlighted in gray, and a dashed line is between bases 20 and 21. (c) The percent of T1 edited alleles versus the inheritance pattern for edits observed for Unique_on, Promiscuous_on, and the pooled off‐target sites. Non‐Mendelian inheritance pattern is classified as edits found in <20% of the T1 progeny from its T0 parent, whereas near‐Mendelian is in <20% if the T1 progeny from its T0 parent.

The genomic regions for all 78 genomic sites predicted to be potential off‐targets in vitro by NoteSeQ were examined for their potential to create viable assays. The off‐target sites for Unique crRNA often fell into low complexity and/or highly repetitive regions of the genome, impeding assay creation. AgriPlex was able to develop assays for 13/39 Unique off‐target genomic loci, comprising 13/22 distinct off‐target sequences. For Promiscuous crRNA, assays could be created for 30/39 off‐target genomic loci, comprising 18/19 distinct target sequences. Of the assayed target sites, editing was not detected at any off‐target sites in Unique plants. Off‐target edits for 12 genomic loci and 4 distinct target sites were found in Promiscuous T1 plants (Figure 5a). The promiscuous_8 locus showed only .14% editing (1 plant) in the T1 generation. This is below the .18% maximum for background variation, so it is unclear whether it represents a true off‐target edit or simply background variation. Of the rest, all but one of the Promiscuous off‐target sites possessing edits had edits in <5% of the total T1 plants, compared with 72%–86% of the total T1 plants for Promiscuous_on and Unique_on sites, respectively. One notable exception was the Promiscuous_3 chr9_10365831 locus, which was edited in 63% of T1 Promiscuous plants. Promiscuous_3 sites had 3 mismatches that were in bases 21–23 relative to the protospacer (Figure 5b). Mismatches in bases 21–23 showed minimal impact on the NoteSeQ score, with Promiscuous_3 and Promiscuous_2 both having scores of >1 (Figure 5b and Table S4). When comparing potential off‐target loci with additional mismatches, such as Promisuous_11 versus Promiscuous_12, there was minimal impact on the NoteSeQ score when additional mismatches were added in the 19–23 range, but in planta data only showed editing at Promiscuous_11 sites. Despite Promiscuous crRNA displaying off‐target activity, edited plants free of off‐target editing could be found in 39/60 (65%) of the original T0 Promiscuous plants compared with 59/59 of the original T0 Unique plants.

## 4 DISCUSSION

In the present study, we examined the editing rates of LbCas12a in soybeans to understand how crRNA design affects both on‐target and off‐target editing in planta. Although multiple aspects of Cas12a editing have been reported for multiple crops, such as rice, Arabidopsis, tomato, and tobacco (Bernabé‐Orts et al., 2019; Tang et al., 2018; Zhang et al., 2023), these have been relatively small‐scale experiments. The work reported here focused on larger plant numbers, following ~60 T0 plants each for two crRNAs through the T1 generation to examine inheritance patterns of on‐target edits and editing at off‐target sites to provide a clearer picture of LbCas12a editing characteristics/patterns in soybean.

The wide variety of inheritance patterns for observed edits suggests a high rate of chimerism in soybean when using a constitutively expressed LbCas12a. For the observed T0 edits, 12% and 23% were not inherited in the T1 generation in plants containing the Unique or Promiscuous crRNAs, respectively. These were likely chimeric edits that occurred in all or part of the unifoliate leaf but were not present in the meristematic tissue that generated the rest of the plant. Correspondingly, non‐inherited edits often had low percent reads in the T0 generation, further supporting their chimeric nature (Figure 4a). On the other end of the spectrum, 45%–50% of the edits observed in T1 were not observed in T0 plants. Because the T1 plants evaluated were null for the LbCas12a transgene, these edits had to have been generated in the T0 plants. Whereas edits inherited from the T0 generation often showed near‐Mendelian inheritance and were on average found in 63%–66% of the T1 progeny for their respective line, these T1 only edits were only found in 19%–25% of the T1 progeny, with 66% and 82% of the T1 only edits having non‐Mendelian inheritance in plants containing the Unique or Promiscuous crRNAs, respectively. The constitutive expression of the LbCas12a gene combined with the sympodial nature of soybean likely led to edits occurring after the formation of the unifoliate leaf that were only present in certain branches, inflorescence, or pods of the T0 plant. Because the seed was harvested and analyzed on a whole plant basis, edits that occur later in the plant's life cycle would not follow the standard Mendelian inheritance pattern because they were only present in certain sections of the plant. Editing chimerism has been observed for many other plant species, such as rice, spruce, apple, and pear (Cui et al., 2021; Ishizaki, 2016; Jang et al., 2016; Malabarba et al., 2020).

Previous studies in rice and Arabidopsis (Bernabé‐Orts et al., 2019; Tang et al., 2018) have reported no detectable off‐target edits with Cas12a using whole genome sequencing on a limited number of plants. The work described here examines the frequency of off‐target edits both in vitro and in planta, monitoring >12,000 genomic locations in vitro by NoteSeQ and 13–39 loci in planta. The Unique crRNA, chosen because it was computationally predicted to have no off‐target editing capability, indeed showed no off‐target activity in any of the 689 plants assayed. In contrast, the Promiscuous crRNA generated off‐target edits in 455/694 plants. Young et al. (2019) saw similar results for Cas9 in maize, where designing guides that were computationally predicted to have low off‐target potential did not show detectable off‐target editing, whereas one designed to be “promiscuous” did. This has also been demonstrated in animal studies with Cas9 (Akcakaya et al., 2018). Together, this demonstrates that proper gRNA/crRNA design can reduce or eliminate off‐target editing for multiple enzymes and multiple species of crops and animals.

Because the location and number of mismatches tolerated can be specific to each enzyme, we next examined the sequences of the in vitro and in planta off‐target sites to determine the common characteristics for LbCas12a editing. Both NoteSeQ and in planta data show that mismatches in bases 21–23 of the protospacer did not inhibit the editing rate because both Promiscous_2 and Promiscuous_3 target sites had scores greater than 1 for NoteSeQ and the chr9_10365831 locus containing Promiscuous_3 site edited at rates similar to the Promiscuous_on locus (Figure 5b). In contrast, mismatches in the first 20 nt of the protospacer decreased NoteSeQ scores by 4–100 fold and the in planta editing rates by 10–>100 fold. Together, this shows that while bases 21–23 of the protospacer are highly tolerant of mismatches, bases 1–20 are not. This mirrors data from Kleinstiver et al. (2016) and Kim et al. (2016) with LbCas12a in human cells, where they showed mismatches in 19–23 nt of the protospacer were tolerated, but mismatches in the first 18 greatly reduced or eliminated editing activity. In addition, Zetsche et al. (2015) observed that crRNAs as short as 18 nt show little to no reduction in activity, indicating that bases 19–23 contribute less to the overall editing activity than the first 18. Thus, when predicting potential off‐target locations bioinformatically, more promiscuity could be allowed in 19–23 nt than in the first 18 nt.

Editing at Promiscuous off‐target sites showed lower rates of inheritance when compared with either the Unique or Promiscuous on‐target sites. Promiscuous off‐target sites had an average of 55% of alleles having non‐Mendelian inheritance patterns, compared with Promiscuous_on site at 45% and Unique_on site at 31% (Figure 5c). This difference is even more striking when the Promiscuous_3 locus chr9_10365831, which edits at near‐on‐target rates, is removed, increasing the percent alleles in the non‐Mendelian category to 70%. The increased chimerism of the Promiscuous off‐target sites, compared with the Unique or Promiscuous on‐target sites, likely reflects the binding kinetics of the enzyme itself. Strohkendl et al. (2018) showed the R‐loop formation is the rate‐limiting step for editing and that a single mismatch in the first 20 nt substantially reduced the k_on_ and increased dissociation kinetics. Stable R‐loop formation required for cleavage at off‐target sites should occur less frequently than at on‐target sites, potentially leading to editing at off‐target sites occurring later in the plant's lifecycle.

CRISPR technology is poised to be a powerful driver of plant breeding and will move from a single gene target to a highly multiplexed system targeting whole gene families and gene networks (Lorenzo et al., 2023; Zhang et al., 2021). This work demonstrated that in addition to edits observed at T0, many edits at on‐ and off‐target sites occur after the initial sampling; therefore, genetic characterization of the plant should be done at the T1 generation after Cas12a has been segregated away. Additionally, the ability to computationally predict and select crRNAs with low off‐target editing potential enables crop editing design and application that facilitate the selection of plants with the intended edits. In summary, the comprehensive off‐target profiling of plants edited with computationally designed “promiscuous” and “unique” crRNAs from the present study showed that: (1) crRNAs designed to have no off‐target potential do not lead to off‐target edits in planta; (2) mismatches in bases 21–23 are tolerated with very little reduction in editing rate; (3) mismatches in the first 20 nt of the crRNA can drastically reduce the editing rate; (4) edits at off‐target sites have lower inheritance rates than on‐target edits. The findings from this study confirm that proper computational design of LbCas12a crRNAs is an effective tool to minimize the impact of off‐target editing in planta (Graham et al., 2020).

## AUTHOR CONTRIBUTIONS

Linda Rymarquis and Miguel Vega‐Sanchez conceptualized the study. Linda Rymarquis and Chenxi Wu wrote the paper. Diane Hohorst managed work between the various teams to get T0 plants produced, T0 and T1 DNA extracted, and assays created. Douglas R. Smith, Vijetha Vemulapalli, and Thomas E. Mullen managed ONE‐seq data generation and analysis.

## CONFLICT OF INTEREST STATEMENT

At the time this manuscript was written, all authors were employees of Bayer Crop Science, a major producer of agricultural seed, or SeQure Dx, a CRISPR off‐target profiling company.

## PEER REVIEW

The peer review history for this article is available in the supporting information for this article.

## Supporting information


**Data S1.** Peer Review.

**Table S1.** Computationally predicted on and off‐targets and editing rates for crRNA targeting the chloroplastic 2‐isopropylmalate synthase 2 gene.

**Table S2.** Primers and probes used to detect editing or conduct copy number assays.

**Table S3.** Kmers used to identify reads specific for each genomic locus.

**Table S4.** in vitro NoteSeq data for targets above background activity.

**Table S5.** Edited alleles observed in T1 progeny.

**Figure S1.** Edit calling for AgriPlex PlexSeq libraries.

## Data Availability

R1 sequencing data is available under Bioproject PRJNA1095968 in the NCBI database.

## References

[pld3627-bib-0001] Akcakaya, P. , Bobbin, M. L. , Guo, J. A. , Malagon‐Lopez, J. , Clement, K. , Garcia, S. P. , Fellows, M. D. , Porritt, M. J. , Firth, M. A. , Carreras, A. , Baccega, T. , Seeliger, F. , Bjursell, M. , Tsai, S. Q. , Nguyen, N. T. , Nitsch, R. , Mayr, L. M. , Pinello, L. , Bohlooly‐Y, M. , … Joung, J. K. (2018). In vivo CRISPR editing with no detectable genome‐wide off‐target mutations. Nature, 561(7723), 416–419. 10.1038/s41586-018-0500-9 30209390 PMC6194229

[pld3627-bib-0002] Aksoy, E. , Yildirim, K. , Kavas, M. , Kayihan, C. , Yerlikaya, B. , Calik, I. , Sevgen, I. , & Demirel, U. (2022). General guidelines for CRISPR/Cas‐based genome editing in plants. Molecular Biology Reports, 49(12), 12151–12164. 10.1007/s11033-022-07773-8 36107373

[pld3627-bib-0003] Asmamaw, M. , & Zawdie, B. (2021). Mechanism and applications of CRISPR/Cas‐9‐mediated genome editing. Biologics., 15, 353–361. 10.2147/BTT.S326422 34456559 PMC8388126

[pld3627-bib-0004] Atkins, A. , Chung, C. H. , Allen, A. G. , Dampier, W. , Gurrola, T. E. , Sariyer, I. K. , Nonnemacher, M. R. , & Wigdahl, B. (2021). Off‐target analysis in gene editing and applications for clinical translation of CRISPR/Cas9 in HIV‐1 therapy. Frontiers in Genome Editing, 3, 673022. 10.3389/fgeed.2021.673022 34713260 PMC8525399

[pld3627-bib-0005] Bernabé‐Orts, J. M. , Casas‐Rodrigo, I. , Minguet, E. G. , Landolfi, V. , Garcia‐Carpintero, V. , Gianoglio, S. , Vázquez‐Vilar, M. , Granell, A. , & Orzaez, D. (2019). Assessment of Cas12a‐mediated gene editing efficiency in plants. Plant Biotechnology Journal, 17(10), 1971–1984. 10.1111/pbi.13113 30950179 PMC6737022

[pld3627-bib-0006] Bolger, A. M. , Lohse, M. , & Usadel, B. (2014). Trimmomatic: A flexible trimmer for Illumina sequence data. Bioinformatics, 30(15), 2114–2120. 10.1093/bioinformatics/btu170 24695404 PMC4103590

[pld3627-bib-0007] Brain, R. , Perkins, D. , Ghebremichael, L. , White, M. , Goodwin, G. , & Aerts, M. (2023). The shrinking land challenge. ACS Agricultural Science & Technology, 3, 152–157. 10.1021/acsagscitech.2c00250

[pld3627-bib-0008] Cui, Y. , Zhao, J. , Gao, Y. , Zhao, R. , Zhang, J. , & Kong, L. (2021). Efficient multi‐sites genome editing and plant regeneration via somatic embryogenesis in *Picea glauca* . Frontiers in Plant Science, 12, 751891. 10.3389/fpls.2021.751891 34721480 PMC8551722

[pld3627-bib-0009] de Kraker, J. W. , Luck, K. , Textor, S. , Tokuhisa, J. G. , & Gershenzon, J. (2007). Two Arabidopsis genes (IPMS1 and IPMS2) encode isopropylmalate synthase, the branchpoint step in the biosynthesis of leucine. Plant Physiology, 143(2), 970–986. 10.1104/pp.106.085555 17189332 PMC1803721

[pld3627-bib-0011] Doench, J. G. , Fusi, N. , Sullender, M. , Hegde, M. , Vaimberg, E. W. , Donovan, K. F. , Smith, I. , Tothova, Z. , Wilen, C. , Orchard, R. , Virgin, H. W. , Listgarten, J. , & Root, D. E. (2016). Optimized sgRNA design to maximize activity and minimize off‐target effects of CRISPR‐Cas9. Nature Biotechnology, 34(2), 184–191. 10.1038/nbt.3437 PMC4744125 26780180

[pld3627-bib-0012] Doench, J. G. , Hartenian, E. , Graham, D. B. , Tothova, Z. , Hegde, M. , Smith, I. , Sullender, M. , Ebert, B. L. , Xavier, R. J. , & Root, D. E. (2014). Rational design of highly active sgRNAs for CRISPR‐Cas9‐mediated gene inactivation. Nature Biotechnology, 32(12), 1262–1267. 10.1038/nbt.3026 PMC4262738 25184501

[pld3627-bib-0014] Frock, R. L. , Hu, J. , Meyers, R. M. , Ho, Y. J. , Kii, E. , & Alt, F. W. (2015). Genome‐wide detection of DNA double‐stranded breaks induced by engineered nucleases. Nature Biotechnology, 33, 179–186. 10.1038/nbt.3101 PMC4320661 25503383

[pld3627-bib-0015] Gerashchenkov, G. A. , Rozhnova, N. A. , Kuluev, B. R. , Kiryanova, O. Y. , Gumerova, G. R. , Knyazev, A. V. , Vershinina, Z. R. , Mikhailova, E. V. , Chemeris, D. A. , Matniyazov, R. T. , Baimiev, A. K. , Gubaidullin, I. M. , Baimiev, A. K. , & Chemeris, A. V. (2020). Design of guide RNA for CRISPR/Cas plant genome editing. Molekuliarnaia Biologiia (Mosk), 54(1), 29–50. 10.31857/S0026898420010061 32163387

[pld3627-bib-0016] Graham, N. , Patil, G. B. , Bubeck, D. M. , Dobert, R. C. , Glenn, K. C. , Gutsche, A. T. , Kumar, S. , Lindbo, J. A. , Maas, L. , May, G. D. , Vega‐Sanchez, M. E. , Stupar, R. M. , & Morrell, P. L. (2020). Plant genome editing and the relevance of off‐target changes. Plant Physiology, 183(4), 1453–1471. 10.1104/pp.19.01194 32457089 PMC7401131

[pld3627-bib-0017] Guo, C. , Ma, X. , Gao, F. , & Guo, Y. (2023). Off‐target effects in CRISPR/Cas9 gene editing. Frontiers in Bioengineering and Biotechnology, 11, 1143157. 10.3389/fbioe.2023.1143157 36970624 PMC10034092

[pld3627-bib-0018] Ishizaki, T. (2016). CRISPR/Cas9 in rice can induce new mutations in later generations, leading to chimerism and unpredicted segregation of the targeted mutation. Mol. Breeding, 36, 165. 10.1007/s11032-016-0591-7

[pld3627-bib-0019] Jang, G. , Lee, S. , Um, T. Y. , Chang, S. , Lee, H. , Chung, P. , Kim, J. , & Choi, Y. (2016). Genetic chimerism of CRISPR/Cas9‐mediated rice mutants. Plant Biotechnology Reports, 10, 425–435. 10.1007/s11816-016-0414-7

[pld3627-bib-0020] Jiménez, C. , & Crosetto, N. (2023). Discovering CRISPR–Cas off‐target breaks. Nature Methods, 20, 641–642. 10.1038/s41592-023-01847-6 37024652

[pld3627-bib-0021] Jinek, M. , Chylinski, K. , Fonfara, I. , Hauer, M. , Doudna, J. A. , & Charpentier, E. (2012). A programmable dual‐RNA‐guided DNA endonuclease in adaptive bacterial immunity. Science, 337(6096), 816–821. 10.1126/science.1225829 22745249 PMC6286148

[pld3627-bib-0022] Jones, S. K. Jr. , Hawkins, J. A. , Johnson, N. V. , Jung, C. , Hu, K. , Rybarski, J. R. , Chen, J. S. , Doudna, J. A. , Press, W. H. , & Finkelstein, I. J. (2021). Massively parallel kinetic profiling of natural and engineered CRISPR nucleases. Nature Biotechnology, 39(1), 84–93. 10.1038/s41587-020-0646-5 PMC9665413 32895548

[pld3627-bib-0054] Kim, D. , Kim, J. , Hur, J. K. , Been, K. W. , Yoon, S. , & Kim, J.‐S. (2016). Genome‐wide analysis reveals specificities of Cpf1 endonucleases in human cells. Nature Biotechnology, 34(8), 863–868. 10.1038/nbt.3609 27272384

[pld3627-bib-0023] Kim, D. , & Kim, J. S. (2018). DIG‐seq: A genome‐wide CRISPR off‐target profiling method using chromatin DNA. Genome Research, 28, 1894–1900. 10.1101/gr.236620.118 30413470 PMC6280750

[pld3627-bib-0024] Kleinstiver, B. P. , Tsai, S. Q. , Prew, M. S. , Nguyen, N. T. , Welch, M. M. , Lopez, J. M. , McCaw, Z. R. , Aryee, M. J. , & Joung, J. K. (2016). Genome‐wide specificities of CRISPR‐Cas Cpf1 nucleases in human cells. Nature Biotechnology, 34(8), 869–874. 10.1038/nbt.3620 PMC4980201 27347757

[pld3627-bib-0025] Kouranov, A. , Armstrong, C. , Shrawat, A. , Sidorov, V. , Huesgen, S. , Lemke, B. , Boyle, T. , Gasper, M. , Lawrence, R. , & Yang, S. (2022). Demonstration of targeted crossovers in hybrid maize using CRISPR technology. Communications Biology, 5(1), 53. 10.1038/s42003-022-03004-9 35027641 PMC8758740

[pld3627-bib-0026] Kuan, P. F. , Powers, S. , He, S. , Li, K. , Zhao, X. , & Huang, B. (2017). A systematic evaluation of nucleotide properties for CRISPR sgRNA design. BMC Bioinformatics, 18(1), 297. 10.1186/s12859-017-1697-6 28587596 PMC5461693

[pld3627-bib-0053] Langmead, B. , Trapnell, C. , Pop, M. , & Salzberg, S. L. (2009). Ultrafast and memory‐efficient alignment of short DNA sequences to the human genome. Genome Biology, 10(3), R25. 10.1186/gb-2009-10-3-r25 19261174 PMC2690996

[pld3627-bib-0028] Li, J. , Manghwar, H. , Sun, L. , Wang, P. , Wang, G. , Sheng, H. , Zhang, J. , Liu, H. , Qin, L. , Rui, H. , Li, B. , Lindsey, K. , Daniell, H. , Jin, S. , & Zhang, X. (2019). Whole genome sequencing reveals rare off‐target mutations and considerable inherent genetic or/and somaclonal variations in CRISPR/Cas9‐edited cotton plants. Plant Biotechnology Journal, 17(5), 858–868. 10.1111/pbi.13020 30291759 PMC6587709

[pld3627-bib-0029] Lorenzo, C. D. , Debray, K. , Herwegh, D. , Develtere, W. , Impens, L. , Schaumont, D. , Vandeputte, W. , Aesaert, S. , Coussens, G. , De Boe, Y. , Demuynck, K. , Van Hautegem, T. , Pauwels, L. , Jacobs, T. B. , Ruttink, T. , Nelissen, H. , & Inzé, D. (2023). BREEDIT: A multiplex genome editing strategy to improve complex quantitative traits in maize. The Plant Cell, 35(1), 218–238. 10.1093/plcell/koac243 36066192 PMC9806654

[pld3627-bib-0030] Malabarba, J. , Chevreau, E. , Dousset, N. , Veillet, F. , Moizan, J. , & Vergne, E. (2020). New strategies to overcome present CRISPR/Cas9 limitations in apple and pear: Efficient dechimerization and base editing. International Journal of Molecular Sciences, 22(1), 319. 10.3390/ijms22010319 33396822 PMC7795782

[pld3627-bib-0031] Malinin, N. L. , Lee, G. , Lazzarotto, C. R. , Li, Y. , Zheng, Z. , Nguyen, N. T. , Liebers, M. , Topkar, V. V. , Iafrate, A. J. , Le, L. P. , Aryee, M. J. , Joung, J. K. , & Tsai, S. Q. (2021). Defining genome‐wide CRISPR‐Cas genome‐editing nuclease activity with GUIDE‐seq. Nature Protocols, 16(12), 5592–5615. 10.1038/s41596-021-00626-x 34773119 PMC9331158

[pld3627-bib-0032] Manghwar, H. , Li, B. , Ding, X. , Hussain, A. , Lindsey, K. , Zhang, X. , & Jin, S. (2020). CRISPR/Cas systems in genome editing: Methodologies and tools for sgRNA design, off‐target evaluation, and strategies to mitigate off‐target effects. Advancement of Science, 7, 1902312. 10.1002/advs.201902312 PMC7080517 32195078

[pld3627-bib-0033] Modrzejewski, D. , Hartung, F. , Lehnert, H. , Sprink, T. , Kohl, C. , Keilwagen, J. , & Wilhelm, R. (2020). Which factors affect the occurrence of off‐target effects caused by the use of CRISPR/Cas: A systematic review in plants. Frontiers in Plant Science, 11, 574959. 10.3389/fpls.2020.574959 33329634 PMC7719684

[pld3627-bib-0034] Modrzejewski, D. , Hartung, F. , Sprink, T. , Krause, D. , Kohl, C. , & Wilhelm, R. (2019). What is the available evidence for the range of applications of genome‐editing as a new tool for plant trait modification and the potential occurrence of associated off‐target effects: A systematic map. Environmental Evidence., 8, 27. 10.1186/s13750-019-0171-5

[pld3627-bib-0035] Naeem, M. , Majeed, S. , Hoque, M. Z. , & Ahmad, I. (2020). Latest developed strategies to minimize the off‐target effects in CRISPR‐Cas‐mediated genome editing. Cells, 9, 1608. 10.3390/cells9071608 32630835 PMC7407193

[pld3627-bib-0036] Park, J. , Bae, S. , & Kim, J.‐S. (2015). Cas‐Designer: A web‐based tool for choice of CRISPR‐Cas9 target sites. Bioinformatics, 31(24), 4014–4016. 10.1093/bioinformatics/btv537 26358729

[pld3627-bib-0037] Pearson, W. , & Lipman, D. (1988). Improved tools for biological sequence analysis. PNAS, 85(8), 2444–2448. 10.1073/pnas.85.8.2444 3162770 PMC280013

[pld3627-bib-0038] Petri, K. , Kim, D. Y. , Sasaki, K. E. , Canver, M. C. , Wang, X. , Shah, H. , Lee, H. , Horng, J. , Clement, K. , Iyer, S. , Garcia, S. , Guo, J. , Newby, G. , Pinello, L. , Liu, D. , Aryee, M. , Musunuru, K. , Joung, J. , & Pattanayak, V. (2021). Global‐scale CRISPR gene editor specificity profiling by ONE‐seq identifies population‐specific, variant off‐target effects. bioRxiv. 2021.04.05.438458. 10.1101/2021.04.05.438458

[pld3627-bib-0039] Sagarbarria, M. , Caraan, J. , & Layos, A. (2023). Usefulness of current sgRNA design guidelines and in vitro cleavage assays for plant CRISPR/Cas genome editing: A case targeting the polyphenol oxidase gene family in eggplant (*Solanum melongena* L.). Transgenic Research, 32(6), 561–573. 10.1007/s11248-023-00371-9 37874448

[pld3627-bib-0042] Strohkendl, I. , Saifuddin, F. A. , Rybarski, J. R. , Finkelstein, I. J. , & Russell, R. (2018). Kinetic basis for DNA target specificity of CRISPR‐Cas12a. Molecular Cell, 71(5), 816–824.e3. 10.1016/j.molcel.2018.06.043 30078724 PMC6679935

[pld3627-bib-0043] Tang, X. , Liu, G. , Zhou, J. , Ren, Q. , You, Q. , Tian, L. , Xin, X. , Zhong, Z. , Liu, B. , Zheng, X. , Zhang, D. , Malzahn, A. , Gong, Z. , Qi, Y. , Zhang, T. , & Zhang, Y. (2018). A large‐scale whole‐genome sequencing analysis reveals highly specific genome editing by both Cas9 and Cpf1 (Cas12a) nucleases in rice. Genome Biology, 19(1), 84. 10.1186/s13059-018-1458-5 29973285 PMC6031188

[pld3627-bib-0044] Tsai, S. Q. , Nguyen, N. T. , Malagon‐Lopez, J. , Topkar, V. V. , Aryee, M. J. , & Joung, J. K. (2017). CIRCLE‐seq: A highly sensitive in vitro screen for genome‐wide CRISPR‐Cas9 nuclease off‐targets. Nature Methods, 14(6), 607–614. 10.1038/nmeth.4278 28459458 PMC5924695

[pld3627-bib-0045] Wang, X. , Tu, M. , Wang, Y. , Yin, W. , Zhang, Y. , Wu, H. , Gu, Y. , Li, Z. , Xi, Z. , & Wang, X. (2021). Whole‐genome sequencing reveals rare off‐target mutations in CRISPR/Cas9‐edited grapevine. Horticulture Research, 8(1), 114. 10.1038/s41438-021-00549-4 33931634 PMC8087786

[pld3627-bib-0047] Ye, X. , Vaghchhipawala, Z. , Williams, E. J. , Fu, C. , Liu, J. , Lu, F. , Hall, E. L. , Guo, S. X. , Frank, L. , & Gilbertson, L. A. (2023). Cre‐mediated autoexcision of selectable marker genes in soybean, cotton, canola and maize transgenic plants. Plant Cell Reports, 42(1), 45–55. 10.1007/s00299-022-02935-1 36316413

[pld3627-bib-0048] Young, J. , Zastrow‐Hayes, G. , Deschamps, S. , Svitashev, S. , Zaremba, M. , Acharya, A. , Paulraj, S. , Peterson‐Burch, B. , Schwartz, C. , Djukanovic, V. , Lenderts, B. , Feigenbutz, L. , Wang, L. , Alarcon, C. , Siksnys, V. , May, G. , Chilcoat, N. D. , & Kumar, S. (2019). CRISPR‐Cas9 editing in maize: Systematic evaluation of off‐target activity and its relevance in crop improvement. Scientific Reports, 9(1), 6729. 10.1038/s41598-019-43141-6 31040331 PMC6491584

[pld3627-bib-0049] Zetsche, B. , Gootenberg, J. S. , Abudayyeh, O. O. , Slaymaker, I. M. , Makarova, K. S. , Essletzbichler, P. , Volz, S. E. , Joung, J. , van der Oost, J. , Regev, A. , Koonin, E. V. , & Zhang, F. (2015). Cpf1 is a single RNA‐guided endonuclease of a class 2 CRISPR‐Cas system. Cell, 163(3), 759–771. 10.1016/j.cell.2015.09.038 26422227 PMC4638220

[pld3627-bib-0050] Zhang, Y. , Ren, Q. , Tang, X. , Liu, S. , Malzahn, A. A. , Zhou, J. , Wang, J. , Yin, D. , Pan, C. , Yuan, M. , Huang, L. , Yang, H. , Zhao, Y. , Fang, Q. , Zheng, X. , Tian, L. , Cheng, Y. , Le, Y. , McCoy, B. , … Qi, Y. (2021). Expanding the scope of plant genome engineering with Cas12a orthologs and highly multiplexable editing systems. Nature Communications, 12(1), 1944. 10.1038/s41467-021-22330-w PMC8007695 33782402

[pld3627-bib-0051] Zhang, Y. , Wu, Y. , Li, G. , Qi, A. , Zhang, Y. , Zhang, T. , & Qi, Y. (2023). Genome‐wide investigation of multiplexed CRISPR‐Cas12a‐mediated editing in rice. Plant Genome, 16(2), e20266. 10.1002/tpg2.20266 36177842 PMC12806924

