# Supplementary material for: Impact of predictive selection of LbCas12a CRISPR RNAs upon on‐ and off‐target editing rates in soybean
Source: Plant Direct. 2024 Aug 16;8(8):e627. doi: 10.1002/pld3.627 (PMC11328349; doi:10.1002/pld3.627)
Supplement: Supplementary file 7 — Figure S1. Edit calling for AgriPlex PlexSeq libraries. [file PLD3-8-e627-s001.pdf]

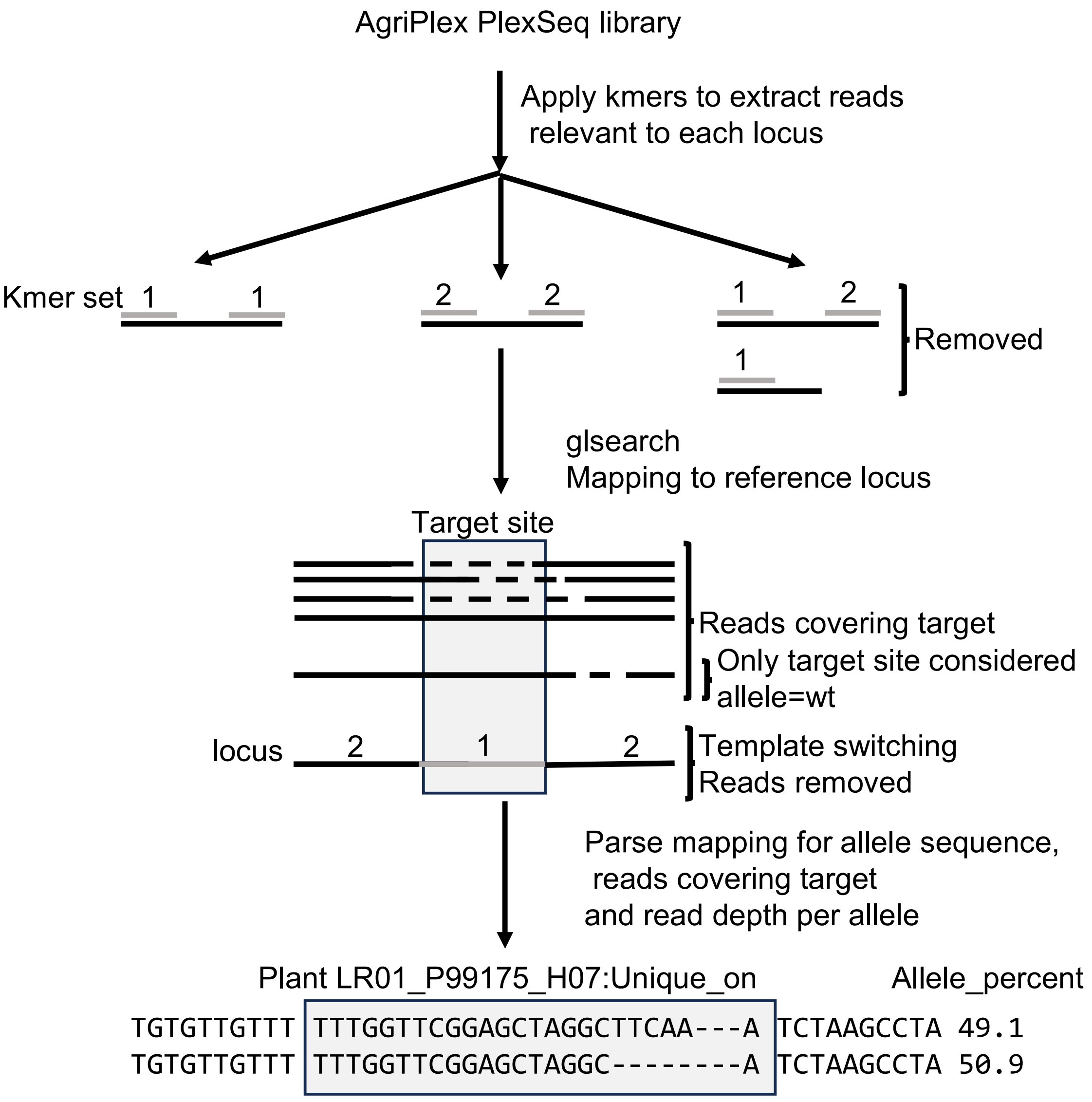

Supplemental Figure 1. Edit calling for AgriPlex PlexSeq libraries. AgriPlex delivered trimmed multiplexed libraries whose sequence was deposited in Bioproject PRJNA1095968. Kmer sets (grey lines, Supplemental table 3) were mapped to reads to demultiplex the library. Reads were mapped against their respective reference locus using glsearch. Mapping data was parsed to calculate the number of reads covering target site (grey box) and identify allele sequences. The dashed lines, representing the indels, that must at least partially overlap the target sequence to be considered a true edit. If an indel existed outside the target region, the allele was characterized by the target site only. Substitutions in the target site consistent with different locus, were considered template switching PCR artifacts and were removed. The number of reads per allele was calculated and the allele percent calculated (reads supporting allele/reads covering target*100). An example of the edited alleles from Supplemental table 5 is show for the Unique_on locus.
