# Supplementary material for: Impact of predictive selection of LbCas12a CRISPR RNAs upon on‐ and off‐target editing rates in soybean
Source: Plant Direct. 2024 Aug 16;8(8):e627. doi: 10.1002/pld3.627 (PMC11328349; doi:10.1002/pld3.627)
Supplement: Supplementary file 1 — Data S1. Peer Review. [file PLD3-8-e627-s006.pdf]

Plant Direct
Open Access

Title: Impact of predictive selection of LbCas12a crRNAs upon on- and off-target editing rates in soybean

Authors

Linda Rymarquis[1*], Chenxi Wu[1], Diane Hohorst[1], Miguel Vega-Sanchez[1], Thomas E. Mullen[2], Vijetha Vemulapalli[2], Douglas R. Smith[2]

Decision Letter Round 1:

June 10, 2024

Dr. Linda Rymarquis
Bayer
Chesterfield, Missouri

RE: Impact of predictive selection of LbCas12a crRNAs upon on- and off-target editing rates in soybean

Dear Dr. Rymarquis:

Thank you for submitting to Plant Direct. All required reviews have been returned and we have now finished our evaluation of your manuscript. In light of the reviewers' and editor's comments, further revisions are needed before the paper can be accepted for publication in Plant Direct.

Please view the editors' and reviewers' comments below and use their suggestions as a guide while you work on your revision. It is important to note that Plant Direct does not require new experiments if there is no technical issue, thus, it may be possible to provide a response to reviewers if you think that an experiment is beyond the scope of the current work.

When uploading the revised version of this article, please be sure to include the following:

-A word document that contains your response to the reviewers. You should respond to each reviewer comment and note the changes made to the manuscript. If you do not agree with a reviewer's comment and choose not to make a suggested revision, please explain why. Please try to provide as complete an answer as possible to each reviewer's criticisms.

-A tracked changes document with each change highlighted
- A clean version of the latest version of the manuscript

To upload your revision, please click the link below.
https://plantdirect.msubmit.net/cgi-bin/main.plex?el=A6Lr6hq5A4omJ1I3A9ftd4zWAAbMUX7sKvoCxul9JgZ

In order to provide as timely a service as possible, we ask that your revision is resubmitted within three months after receipt of this request. If an extension is needed, please send a request, along with a brief explanation, to the editorial office at plantdirect@wiley.com .

Please note that, in addition to publishing reviewer comments, the author's responses to review comments will also be published alongside the final version of the paper. If you would not like the author's responses to be published, please contact the editorial office at plantdirect@wiley.com .

Thank you very much for giving us an opportunity to review your work. I look forward to receiving the next version.

Sincerely,

Gustavo MacIntosh

Editor, Plant Direct

-- Reviewer comments:
Reviewer #1:

This manuscript reports an industrial-scale evaluation of soybean mutant lines generated by CRISPR-mediated mutagenesis using LbCas12a. This research compared hundreds of soybean lines resulting from using either a unique crRNA or a promiscuous crRNA for their on-target

efficiency and off-target rates. While not wholly a novel concept or approach, this type of data is sorely needed by the community not only for the improvement of the editing tools but also the consumer confidence in edited ag products. Too often, academic research groups do not have the resources to generate large data sets. The reviewer commends the willingness of the private laboratory to share their research outcomes with the community.

1. Figure 1: The alignment of two sequences, "Promiscuous" and "Unique," needs to be adjusted. Currently, printout from the PDF file does not really make sense. Please align the two sequences. Please use the appropriate font type in which each letter has the same width. The most commonly used font type for sequence display is "Courier".

2. Figure 1: Please make sure each feature is adequately described in the figure legends. For example, what do rectangle boxes, triangle boxes, and an arrow stand for?

3. Figure 1: tDNA should be T-DNA

4. Supplemental files. This reviewer appreciates that the authors disclose all detailed sequences raw data in the manuscript. This reviewer has never seen a supplemental file of over 13300 pages in the past. However, the reviewer does not think this type of information is helpful or useful for readers in the current format. It should be deposited to a public database, but I am not sure where.

Reviewer #2:

The manuscript presents a large-scale study of on- and off-targeted editing and their heritability. Although similar studies were reported, but most of the studies were conducted in a small scale. The study should provide a more comprehensive and reliable knowledge of off-target editing in soybean. The result should be valuable for scientists to design guide RNAs and predict the on- and off-target editing.
Please address the following concerns:
1. Please revise the writing of Result. The introduction and Discussion are well written, but results have several long sentences hard to follow.
2. Please provide whole genome sequencing of at least two T1 plants from unique and promiscuous crRNAs and use the sequencing data to validate the results. Some off-target edited alleles are potentially missed in bioinformatic prediction and In vitro assay.
3. Can Agriplex genotyping technology detect all off-target editing? Particular for large deletion. If not, what percentage of edited alleles can be detected by Agriplex genotyping technology.

How do you handle the edited alleles that Agriplex cannot develop assay for?
4. Line105: "Bins predicted to have >5 target sites that were targeted by a combination of crRNAs with no predicted off-targets and >10 off-target sites were overlapped with gene models to identify those in non-essential/duplicated genes", What is the reason to only test the off-target seq in genes, and how do you select the non-essential genes?
5. Line 139: an Arabidopsis actin promoter: which actin gene promoter?
6. Line 170: "A small amount of endosperm tissue was collected from each seed into 96 well plates". I believe that the authors refer to cotyledon. Does assayed tissue include seed coat? If yes, seed coat is maternal tissue, not identical to cotyledon and plant tissues germinated from the seeds in genetics.
7. Line 248: "Between 77-88% of the edits observed in the T0 generation were inherited in the T1 generation (Sup table 7). The percent reads aligning to a particular edited allele in the T0 assay was predictive of its T1 inheritance.". Are percent reads for on-target editing or off-targeting. It is not clear. Please revise and clearly indicate weather heritability is referred to on-target editing or off-target editing sites.

Reviewer #3:

This is a comprehensive work detailing on- and off-target editing rates in soybean and this study contributes to the present knowledge gene editing in crops in general.

In this manuscript, the authors describe LbCas12a CRSPR editing efficiency in soybean. Initially, in vitro "discovery" step was performed using NoteSeQ, which identified number of unique and promiscuous targets genome wide. Based on their genomic search for unique or promiscuous crRNA targets, they have selected two crRNA targets in chloroplastic 2-isopropylmalate synthase 2 for large scale editing efficiency in-planta. They used targeted amplicon sequencing to analyze the editing efficiency in T0 and T1 generation. Further they have described how to optimize crRNA designing that avoids off-target activities. This is a comprehensive work detailing on- and off-target editing rates in soybean and this study contributes to the present knowledge gene editing in crops in general. Minor comments Although there are data details on the sequencing, it would be good to have some images for the plant samples that were used. Line 213. ‘Six crRNAs targeting chloroplastic 2-isopropylmalate synthase 2, corresponding to 2’ explain why this gene was selected.

Decision Letter Round 2:

July 20, 2024
Dr. Linda Rymarquis

Plant Direct
Open Access

Bayer
Chesterfield, Missouri

MSID: 2024-01504R1
MS TITLE: Impact of predictive selection of LbCas12a crRNAs upon on- and off-target editing rates in soybean

Dear Dr. Linda Rymarquis:

I am pleased to inform you that your manuscript "Impact of predictive selection of LbCas12a crRNAs upon on- and off-target editing rates in soybean" has been accepted for publication in Plant Direct.

Your article will appear online in the next available issue of Plant Direct. To ensure your article gets published as quickly as possible, please pay attention to the steps detailed below. We have found that most of the delays happen at this stage, especially at the payment stage, so please respond as quickly as possible when prompted.

License Agreement: Once your article has been accepted it will move to Production and undergo admin and file checking - you may receive an email with any queries we have at this stage. When all required items are received by the publisher and queries resolved, the corresponding author will receive an email from Wiley's Author Services system which will ask them to log on at https://authorservices.wiley.com/bauthor and will present them with the appropriate license for completion. Your article cannot be published until both the signed license agreement and payment of the article fee have been received.

Payment of the Open Access Article Publication Fee: All articles published in Plant Direct are fully open access: immediately and freely available to read, download and share. Plant Direct charges a publication fee to cover the publication costs. The corresponding author for this manuscript should have already received a quote from the payments team (cs-openaccess@wiley.com) with the estimated article publication fee; please email cs-openaccess@wiley.com if this has not been received. The corresponding author should log on to the Wiley Author Services site, where the publication fee can be paid by credit card or an invoice. Pro Forma can also be requested. Payment of the publication charge must be received before the article will be published online.

Proofs: You will have the opportunity to look over your paper once more when you receive the author proofs for your article. The proofs will be with you in approximately two weeks. Please

note that, in addition to publishing reviewer comments, the author's responses to review comments will also be published alongside the final version of the paper. If you would not like the author's responses to be published, please contact the editorial office at plantdirect@wiley.com .

Promotion of your article: You can help your research get the attention it deserves! Check out Wiley's free Promotion Guide for best-practice recommendations for promoting your work at www.wileyauthors.com/eeo/guide. And learn more about Wiley Editing Services which offers professional video, design, and writing services to create shareable video abstracts, infographics, conference posters, lay summaries, and research news stories for your research at www.wileyauthors.com/eeo/promotion.

Thank you again for your contribution to Plant Direct. If you have any questions, feel free to contact the editorial office at plantdirect@wiley.com .

Sincerely,

Gustavo MacIntosh

Gustavo MacIntosh

Editor, Plant Direct

Author's Response Round 1:

To the reviewers,

Thank you for taking the time to review our paper and provide insightful comments. Our responses to your comments are in green below.

Reviewer #1:

1. Figure 1: The alignment of two sequences, "Promiscuous" and "Unique," needs to be adjusted. Currently, printout from the PDF file does not really make sense. Please align the two sequences. Please use the appropriate font type in which each letter has the same width. The most commonly used font type for sequence display is "Courier".

Figure 1 and caption were modified to make it clear that the two gRNAs do not overlap in the gene sequence. Lines were drawn to indicate that the entire crRNA target site is within the white triangle. In addition, the Promiscuous_on sequence was moved to the top of the gene model to visually separate the two sequences for additional clarity.

2. Figure 1: Please make sure each feature is adequately described in the figure legends. For example, what do rectangle boxes, triangle boxes, and an arrow stand for?

More details were added to Figure 1 caption. It now states, Figure 1. Experimental overview for on and off-target analysis. *In vitro* and *in planta* experiments were done in parallel. The chloroplastic 2-isopropylmalate synthase 2 gene is shown in the 5' to 3' orientation. The black boxes represent the exon, while the white triangles indicate the location and orientation of the Promiscuous_on and Unique_on target sites. The PAM sequences are underlined and bolded. Plants were transformed with a plasmid expressing the LbCas12a nuclease and either the Unique, Promiscuous or no crRNA. The NoteSeQ experiments informed the assay panel for off-target analysis.

3. Figure 1: tDNA should be T-DNA

Changed

4. Supplemental files. This reviewer appreciates that the authors disclose all detailed sequences raw data in the manuscript. This reviewer has never seen a supplemental file of over 13300 pages in the past. However, the reviewer does not think this type of information is helpful or useful for readers in the current format. It should be deposited to a public database, but I am not sure where.

Since the sequence files for the individual plants are publicly available in NCBI, supplemental files 5 and 6 have been removed and sequences of the edited alleles added to the old supplemental file 7. This removes redundancy to cut down on page number, yet still provides the sequences of the edited alleles. The old Sup table 7 was renamed Sup table 5 and the manuscript was edited to match.

Reviewer #2:

1. Please revise the writing of Result. The introduction and Discussion are well written, but results have several long sentences hard to follow.

Edited for clarity and to shorten the sentence length.

2. Please provide whole genome sequencing of at least two T1 plants from unique and promiscuous crRNAs and use the sequencing data to validate the results. Some off-target edited alleles are potentially missed in bioinformatic prediction and In vitro assay.

Off-target editing profiling can be performed using both biased and unbiased methods. As pointed out by a review paper by Sturme et al. 2022, off-target edits were predominantly detected via biased analysis of predicted off-target sites instead of unbiased genome-wide analysis in previous studies. Our method is similar to the one published in maize, which claims that bioinformatic selection of unique target sites can be used as a reliable tool to mitigate the potential for off-target editing in crop plants with reference genomes (Young et al., 2019). Additionally, previous unbiased studies that did use whole genome sequencing (WGS) (Tang et al., 2018; Li et al. 2019; Bernabé-Orts et al., 2019; Zhang et al., 2023), have reported that sequence variations they found were consistent with somaclonal variation, sequencing errors, random mutation, and inherent genetic variation rather than Cas nuclease activity. Because of this, we thought the best way to capture off-target activity was to look at a large number of plants to capture rate off-target edits rather than WGS on just 2 plants. This also provides population level rates for off-targeting rather than just reporting no off-target edits like previous studies have. From a practical standpoint, all the DNA was used for the AgriPlex PlexSeq libraries and is no longer available for these plants, so follow-up WGS is, unfortunately, not possible.

Sturme MHJ, Jan Pieter van der Berg, Lianne M. S. Bouwman, Adinda De Schrijver, Ruud A. de Maagd, Gijs A. Kleter, and Evy Battaglia-de Wilde. Occurrence and Nature of Off-Target Modifications by CRISPR-Cas Genome Editing in Plants. ACS Agricultural Science & Technology 2022 2 (2), 192-20. DOI: 10.1021/acsagscitech.1c00270

Bernabé-Orts, J.M., Casas-Rodrigo, I., Minguet, E.G., Landolfi, V., Garcia-Carpintero, V., Gianoglio, S., Vázquez-Vilar, M., Granell, A., Orzaez, D. (2019) Assessment of Cas12a-mediated gene editing efficiency in plants. *Plant Biotechnol J.* 17(10), 1971-1984. doi: 10.1111/pbi.13113.

Li, J.; Manghwar, H.; Sun, L.; Wang, P.; Wang, G.; Sheng, H.;Zhang, J.; Liu, H.; Qin, L.; Rui, H.; Li, B.; Lindsey, K.; Daniell, H.; Jin,S.; Zhang, X. Whole genome sequencing reveals rare off-target mutations and considerable inherent genetic or/and somaclonal variations in CRISPR/Cas9-edited cotton plants. Plant BiotechnologyJournal 2019, 17 (5), 858−868

Tang, X., Liu, G., Zhou, J., Ren, Q., You, Q., Tian, L., Xin, X., Zhong, Z., Liu, B., Zheng, X., Zhang, D., Malzahn, A., Gong, Z., Qi, Y., Zhang, T., Zhang, Y. (2018) A large-scale whole-genome sequencing analysis reveals highly specific genome editing by both Cas9 and Cpf1 (Cas12a) nucleases in rice. *Genome Biol*. 19(1), 84. doi: 10.1186/s13059-018-1458-5.

Young, J.; Zastrow-Hayes, G.; Deschamps, S.; Svitashev, S.;Zaremba, M.; Acharya, A.; Paulraj, S.; Peterson-Burch, B.; Schwartz,C.; Djukanovic, V.; Lenderts, B.; Feigenbutz, L.; Wang, L.; Alarcon,C.; Siksnys, V.; May, G.; Chilcoat, N. D.; Kumar, S. CRISPR-Cas9 Editing in Maize: Systematic Evaluation of Off-target Activity and ItsRelevance in Crop Improvement. Sci. Rep. 2019, 9 (1), 6729.

Zhang, Y., Wu, Y., Li, G., Qi, A., Zhang, Y., Zhang, T., Qi, Y. (2023) Genome-wide investigation of multiplexed CRISPR-Cas12a-mediated editing in rice. *Plant Genome*. 16(2), e20266. doi: 10.1002/tpg2.20266.

3. Can Agriplex genotyping technology detect all off-target editing? Particular for large deletion. If not, what percentage of edited alleles can be detected by Agriplex genotyping technology. How do you handle the edited alleles that Agriplex cannot develop assay for?

This experiment was designed to get an overview of the off-targeting editing rate on a population level rather than trying to capture every off-target edit within a single plant. AgriPlex PlexSeq detected deletions ranging from 0-46 nt in the T1 plants. This size range represented >90% of the total (Promiscuous +Unique) edits observed in the T0 generation. In addition, out of the 638 T0 plants showing edits, <2% only had edits >50 nt and may have been miscalled if we had used PlesSeq to assay the T0 generation. Thus, it is highly likely that with the large number of plants tested that a site that shows off-targeting activity would have generated at least 1 plant with a smaller deletion in addition to a large deletion. If trying to bring a product to market, I would not recommend using AgriPlex PlexSeq due to its inability to detect large deletions or more complex edits such as inversions or translocation that may occur when more than one crRNA is expressed in a plant (Zhang et al., 2023). For large-scale surveys like this one, it can be a useful and cost-effective tool if all you need is an overview and can tolerate some data loss due to assay difficulties.

4. Line105: "Bins predicted to have >5 target sites that were targeted by a combination of crRNAs with no predicted off-targets and >10 off-target sites were overlapped with gene models to identify those in non-essential/duplicated genes", What is the reason to only test the off-target seq in genes, and how do you select the non-essential genes?

Only the initial selection of the Promiscuous and Unique crRNA was limited to genic regions. The off-target analysis and testing were genome-wide regardless of whether they

were in a genic region or not. The chloroplastic 2-isopropylmalate synthase 2 gene was chosen because it is part of a gene family having at least 4 copies with another 8 genes being listed as probable 2-isopropylmalate synthase in the NCBI database. In addition, de Kraker et al (2007) showed that knocking out 1 copy of the gene in Arabidopsis did not impact plant health. This information was added to the method section of the paper.

de Kraker, J.W., Luck, K., Textor, S., Tokuhisa, J.G., Gershenzon, J. (2007) Two Arabidopsis genes (IPMS1 and IPMS2) encode isopropylmalate synthase, the branchpoint step in the biosynthesis of leucine. Plant Physiol. 143(2), 970-986. doi: 10.1104/pp.106.085555.

5. Line 139: an Arabidopsis actin promoter: which actin gene promoter?

Actin 7. Paper edited to reflect this.

6. Line 170: "A small amount of endosperm tissue was collected from each seed into 96 well plates". I believe that the authors refer to cotyledon. Does assayed tissue include seed coat? If yes, seed coat is maternal tissue, not identical to cotyledon and plant tissues germinated from the seeds in genetics.

Changed to cotyledon, the protocol was originally written for corn where an endosperm sample is taken. The sample would have contained some seed coat in addition to cotyledon tissue, but extensive testing has showed that the seed coat DNA is such a small fraction compared to cotyledon that it does not contribute significantly to the overall genotype. In addition, since we were testing for the lack of the transgene, the presence of any maternal tissue would only lead to a false positive for the presence of the transgene, which would have resulted in the seed not being planted. All editing data was obtained from leaf tissue.

7. Line 248: "Between 77-88% of the edits observed in the T0 generation were inherited in the T1 generation (Sup table 7). The percent reads aligning to a particular edited allele in the T0 assay was predictive of its T1 inheritance.". Are percent reads for on-target editing or off-targeting. It is not clear. Please revise and clearly indicate weather heritability is referred to on-target editing or off-target editing sites.

Only on-target edits were examined at T0 the generation and the text was changed to The percent reads aligning to a particular edited allele in the T0 assay was predictive of its T1 inheritance.

Reviewer 3

Line 213. 'Six crRNAs targeting chloroplastic2-isopropylmalate synthase 2, corresponding to 2' explain why this gene was selected.

The goal was to choose a locus that would not cause any phenotypic difference in the plants when edited to avoid any accidental selection bias during the plant transformation process. The chloroplastic 2-isopropylmalate synthase 2 gene was chosen because it is part of a gene family having at least 4 copies with another 8 genes being listed as probable 2-isopropylmalate synthase in the NCBI database. In addition, de Kraker et al (2007) showed that knocking out 1 copy of the gene in Arabidopsis did not impact plant health. This gene also contained a variety of target sites that had >10 off-targets and no predicted off-targets, meaning we had a better chance of finding 2 that cut at a similar rate to move forward with the large-scale testing.

Although there are data details on the sequencing, it would be good to have some images for the plant samples that were used.

I added Supplemental Figure 1 detailing the analysis of the sequences we obtained from AgriPlex and added more detail to the corresponding paragraph in the paper. The junction sequences of all T1 edited alleles in the experiment can be found in the new Sup table 5.
